# Empathy alleviates the learning burnout of medical college students through enhancing resilience

**DOI:** 10.1186/s12909-022-03554-w

**Published:** 2022-06-20

**Authors:** Wenzhi Wu, Xiao Ma, Yilin Liu, Qiqi Qi, Zhichao Guo, Shujun Li, Lei Yu, Qing Long, Yatang Chen, Zhaowei Teng, Xiujuan Li, Yong Zeng

**Affiliations:** 1 The Sixth Affiliated Hospital of Kunming Medical University, Yuxi, China; 2 The Second Affiliated Hospital of Kunming Medical University, Kunming, China; 3 School of Maxism, Kunming Medical University, Kunming, China; 4 The First People’s Hospital of Yunnan Province, Kunming, China; 5 Department of Psychiatry, Shenzhen Mental Health Center, Shenzhen, China

**Keywords:** Empathy, Learning burnout, Resilience, Medical college students

## Abstract

**Objective:**

The problem of learning burnout of medical students is becoming prominent, and empathy can play a good predictive role in learning burnout. The present study aimed to investigate the relationship between empathy and learning burnout, as well as the mediation effect of resilience in this relation.

**Methods:**

Five hundred and eighty-eighth college students from a key medical university in Yunnan Province was investigated using the Basic Empathy Scale, Learning Burnout Scale, and Connor-Davidson Resilience Scale. All the measures showed good reliability and validity in the present study. Data were analyzed using SPSS 23.0 and Amos 22.0.

**Results:**

Using structural equation modeling, we tested a conceptual model indicated that: (1) medical students' empathy negatively and significantly predicted learning burnout; (2) medical students' empathy positively predicts mental resilience; (3) resilience of medical students negatively predicts learning burnout; (4) resilience partially mediated the relationship between empathy and learning burnout of medical students, while also controlling for family socioeconomic status.

**Conclusion:**

These findings highlight the mediating role of resilience in the effect of empathy on learning burnout of medical college students. It may contribute to a better understanding of the effect of empathy. Moreover, it can also provide constructive suggestions for protecting and improve empathy and resilience of medical college students.

## Introduction

Learning burnout, among other things, refers to the negative physical and psychological performance of students due to over-study [1]. Learning burnout not only affects students' academic performance, but also adversely affects students' physical and mental health and future development, directly leading to a serious decline in academic quality, and thus some students have negative life events such as suspension or dropout [2]. Compared with students from other universities/other subjects, medical school students have a long professional academic system, complex learning content, and high learning pressure, so their learning psychological state should be paid more attention to. A number of studies have shown that medical students show a higher level of learning burnout, and the burnout level improves as the grade increases [3–6]. Learning burnout is an important obstacle to students' learning, which reduces the sense of meaning of learning and causes psychological symptoms such as irritability, tension, low self-esteem and depression, which negatively affects their physical and mental health and personal development [7, 8]. Consequently, learning burnout not only directly affects medical students' learning engagement and academic achievement [9, 10], but also is closely related to their professional satisfaction and professional commitment [11], which will cause some students to fail to achieve the standard of a qualified doctor, which in turn affects the quality of the future medical workforce [12, 13]. Thus, it is particularly important to detect learning burnout early and take measures to effectively prevent and intervene, so as to promote the development of medical students' mental health.

Related studies have shown that empathy can play a good predictive role in learning burnout [1, 14–16]. Empathy refers to the individual's ability to understand and share the emotional experience of others, and to respond appropriately to it. It is generally considered to contain two components: cognitive empathy and emotional empathy [17–20]. Studies have found that there is a negative correlation between empathy and learning burnout in medical students [21, 22], but some studies have reached the opposite conclusion [1, 23], thus the specific mechanism of action of these two variables is not yet clear. Systematic reviews had investigated the effect of empathy of doctors and nurses on job burnout [24, 25], but there was a lack of empirical research on the relationship between empathy and learning burnout in medical students. Cultivating empathy is not only important for medical students to maintain their personality, promote physical and mental health, and improve professionalism, but also help improve their sense of work accomplishment in the future medical practice [26] and build harmony doctor-patient relationship [27]. Therefore, it is necessary to explore the mechanism of empathy and learning burnout, in order to reduce learning burnout through cultivating the empathy ability and professional identity of medical students which would stimulate the students’ enthusiasm for learning, improve their academic level and alleviate learning burnout while.

Resilience refers to the psychological process that an individual can still actively adapt when encountering a major threat or trauma [28]. It is regarded as a good personal quality that can perform well after experiencing setbacks, and it plays an important role in the process of coping with external pressure situations [29–31]. A large number of studies have shown that empathy fatigue is common among medical staffs [32, 33]. Improving psychological resilience can effectively enhance the quality of individual life and mental health status [34], thereby alleviating the impact of empathy fatigue on psychological problems [35]. Empathy and resilience can predict the professional decision-making self-efficacy of individuals to a certain extent, and then affect their work engagement [36]. The improvement of one's own empathy ability can not only improve their psychological resilience level [37, 38], but also promote the generation of pro-social behavior, which is beneficial to the physical and mental health of patients [27, 39–41].

At present, medical students generally have high learning burnout, high psychological pressure, high loneliness, and decreased mental flexibility [42–44]. Resilience has a direct predictive effect on the individual's autonomous learning ability, and it is the main factor that affects the professional applicability and learning career adaptation of medical students [45, 46]. Improving psychological resilience can reduce the generation of learning burnout [30], which was also supported by evidence from multiple systematic reviews [47–49]. The framework of resilience in action points out that the innate potential of individuals is the source of resilience, and external protective factors from society schools, families, relatives and peers can help young people develop individual internal resources, including empathy, cooperation, problem solving and so on, which will protect young people from risk factors and promote their healthy development [50]. Therefore, the improvement of the empathy ability of medical students can help improve the psychological resilience, and a good psychological resilience can relieve the level of learning burnout [14, 51]. In other words, psychological resilience plays a mediating role between empathy and learning burnout in medical students.

In addition, according to the family investment model, families with higher family socioeconomic status (FSES) have significant advantages in terms of income, social capital, and human capital, which can be converted into student development resources, thereby affect the development level of students [52], improve empathy and mental flexibility, and then face learning tasks with a more positive attitude, alleviating learning burnout [53].

In summary, the present study aims to investigate the influence of empathy of medical students on learning burnout and the mediating role of resilience. Therefore, this study takes family socioeconomic status as a control variable. Based on this, this study puts forward the following hypotheses: (1) Medical students' empathy negatively predicts learning burnout (H1), higher empathy ability is beneficial to alleviate learning burnout; (2) Medical students' empathy positively predicts mental resilience (H2); (3) Resilience of medical students negatively predicts learning burnout (H3); (4) Resilience plays a mediating role between empathy and learning burnout of medical students (H4).

## Materials and methods

### Participants

Questionnaires were distributed to 600 medical college students at A key medical university of Yunnan Province in the Southwest of PRC. Excluding invalid questionnaires, a total of 588 samples (362 girls) were collected, with a response rate of 98%. Participants ranged in age from 17 to 26 years (*M* = 19.90 year; *SD* = 1.55 year) and were distributed across the school years as follows: 1st year: *N* = 150; 2nd year: *N* = 290; 3rd year: *N* = 90; 4th year and above: *N* = 58. Participants obtained informed consent before the survey and were rewarded with small gifts such as pencils and erasers after completing the questionnaire.

To test the adequacy of the sample size, the present study used G*Power 3.1 Software for Post-hoc Statistical Efficacy Test (effect size = 0. 15, α = 0.05), the results showed power = 1, indicating sufficient sample size [54].

### Instruments and procedure

Students completed three self-report instruments measuring their empathy, resilience, and learning burnout, respectively. All instruments were in written format and were anonymously administered in the classroom in counterbalanced order. The testing session lasted approximately 15 min.

#### Basic empathy scale

The Basic Empathy Scale consists of 16 items and measures two aspects of empathy abilities: (1) cognitive empathy (referring to the ability to understand other people's emotions, know how others feel, e.g. “When others are happy, I can generally perceive”), (2) emotional empathy (referring to the ability to be emotionally infected by others, e.g. “My friend's mood doesn't affect me much”). The questionnaire uses a five-point Likert scale ranging from 1 (completely disagree) to 5 (completely agree). Responses across the 16 items were summed to obtain the total score, with higher scores indicating higher capacity for empathy [55]. Participants were administered the Chinese version of the instrument which has also been confirmed to have a two-factor structure [56].

#### Connor-Davidson Resilience Scale

The Connor-Davidson Resilience Scale consists of 25 items and measures three aspects of resilience: (1) tenacity (refers to a person's composure, alertness, perseverance and sense of control in the face of difficulties and challenges, e.g. “I know where to go for help”), (2) strength (refers to an individual's recovery after setbacks and past experiences and the ability to become strong, e.g. “Coping with stress makes me feel empowered”), (3) optimism (reflects the individual's tendency to see the positive side of things and trust their own personal and social resources, e.g. “I can cope no matter what happens”). The questionnaire uses a five-point Likert scale ranging from 1 (completely disagree) to 5 (completely agree). Responses across the 25 items were summed to obtain the total score, with higher scores indicating higher capacity for resilience [57]. Studies have proved that it has good reliability and validity in the context of Chinese culture [58].

#### Learning Burnout Scale

The Learning Burnout Scale (LBS) consists of 20 items including three dimensions: (1) rejection (reflects that college students are unable to deal with the problems and requirements in their studies well, e.g. “I feel exhausted after studying all day”), (2) improper behavior strength (reflects that college students are tired of studying thus behaving characteristics such as skipping class, not attending class, being late, leaving early, not handing in homework, etc. e.g. “I seldom study after class”), (3) reduced personal accomplishment (reflecting college students’ feelings of low achievement in the learning process, e.g. “The mastery of professional knowledge is easy for me”). The questionnaire uses a five-point Likert scale ranging from 1 (completely disagree) to 5 (completely agree). Responses across the 20 items were summed to obtain the total score, with higher scores indicating higher degree of learning burnout [59].

#### Family socio-economic status

The family socio-economic status index is composed of family income, parents' education level and occupation. Among them, family income refers to family monthly income. Combined with the actual situation of local economy, the family monthly income is divided into five levels: "less than 2000 yuan", "2000–6000 yuan", "6000–10,000 yuan", "10,000–14,000 yuan" and "more than 14,000 yuan", The education level of parents includes "primary school and below", "junior high school", "senior high school or technical secondary school", "College", "undergraduate" and "graduate". The evaluation method of parents' occupation is based on the five economic and social levels divided by Gu Honglei [60]. The measurement standard of FSES is that the scores of family income, parents' education level and parents' occupation are transformed into standard scores and summed [60, 61].

### Data analysis and conceptual model

**Fig. 1:**
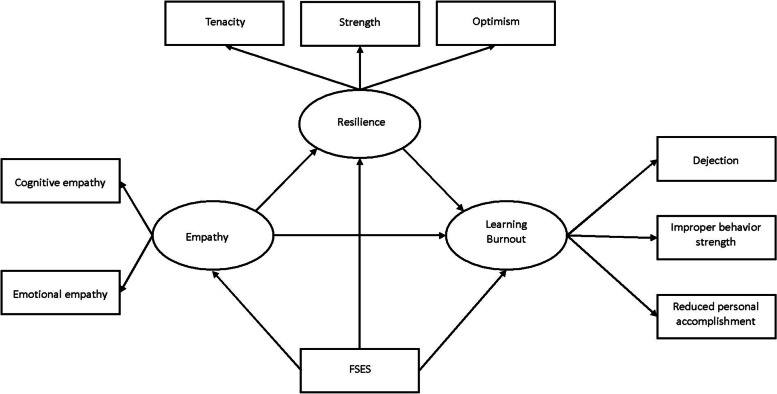
Hypothesized conceptual model of association among empathy, resilience and learning burnout

To identify patterns of association between the participants’ empathy, resilience, and learning burnout, we chose structural equation modeling (SEM) as our method of analysis. SEM methods are a well-established approach to multivariate data analysis that provide robust estimations of the associations among complex variables [62, 63]. In the present study, the proposed conceptual model (see Fig. 1) featured three latent endogenous variables: empathy, resilience, and learning burnout.

Each variable was operationalized via at least two different empirical indicators as the minimum condition for model identification [64]. The total and direct effects of empathy on resilience and learning burnout were estimated within a single structural model, with FSES as an external controlling variable. Mahalanobis’ distances (*p* < 0.001) were computed for all variables, with a view to identifying and, if appropriate, removing multivariate outliers. No extreme multivariate values were found. Descriptive statistics and zero-order correlations were calculated. Next, the data were assessed for normality by computing kurtosis and skewness scores. Given that none of the variables under study had indicators falling outside the recommended thresholds of + 1 and − 1 (see Table 2), the maximum likelihood method was used to estimate the parameters for the structural models [65].

With respect to goodness of fit, two classes of indexes (i.e., statistical indicators reflecting the degree of fit between the hypothesized conceptual model and the empirical data) were adopted: absolute fit and relative fit measures. The former included *χ*^*2*^ and normed-*χ*^*2*^ (NC), where a non-statistically-significant *χ*^*2*^ value and NC values of under 3.0 indicate good fit [62, 66]. The latter comprised comparative fit index (*CFI*), Goodness of Fit Index (*GFI*), normed fit index (*NFI*), Tucker-Lewis Coefficient (*TLI*) and the root mean square error of approximation (*RMSEA*). Thresholds for good model fit were: *CFI* > 0.90, *GFI* > 0.90, *NFI* > 0.90, *TLI* > 0.90, *RMSEA* < 0.08 [67, 68]. To estimate the statistical significance of effects, confidence limits were calculated using both Monte Carlo simulation [69] and bootstrapping methods with a set of random samples (*k* = 2000). Statistical significance test level α = 0. 05, ^*^p < 0.05, ^**^p < 0.01, ^***^p < 0.001.

## Results

### Missing value handling

In the specific data processing process, there are some missing values in the study due to subjects' filling in and answering, data entry and other reasons. The study uses the Little test with completely random missing values to investigate the randomness of missing values. The results show that *χ*^*2*^ (6342) = 14,127.27, *p* < 0.001, indicating that the missing value is not completely random. Therefore, we use Mean Adjusted Maximum Likelihood Estimator (MLM) to deal with missing values.

### Multicollinearity assessment and common method deviation test

The tolerance range of all predictors was 0.95 ~ 0.97 (≤ 0.1 means multicollinearity), and the variance expansion factor was 1.03 ~ 1.05 (≥ 10 means multicollinearity), indicating that there was no multicollinearity problem between predictors.

The common method deviation was controlled by anonymous, different measurement forms and scoring methods, and two methods were used to test the common method deviation. (1) Carry out common variance analysis on the four questionnaires through factor analysis. The chi-square statistic of Bartlett's sphericity test was significant (*χ*^*2*^ (1830) = 14,671.64, *p* < 0.001). After principal component analysis, 18 eigenvalues ​​greater than 1 were extracted. The first factor explaining the variance is 18.622%, which was lower than the 40% required by the critical standard [70], indicating that the questionnaire used in this study did not have a significant common method deviation problem. (2) A four-factor model consisting of four latent variables and their indicators was tested, and the results showed that the four-factor model fits well (*χ*^*2*^ = 110.27, *df* = 20, *CFI* = 0.94, *NFI* = 0.93, *RMSEA* = 0.09). Then, the single-method latent factor method [71, 72] was used to test the five-factor model after adding method factors, and the fitting index was good (*χ*^*2*^ = 72.14, *df* = 14, *CFI* = 0.96, *NFI* = 0.95, *RMSEA* = 0.08). The comparison between the four-factor model and the five-factor model shows that *Δχ*^*2*^ was significant (*Δdf* = 6, *Δχ*^*2*^ = 38.13, *p* < 0.001), but *Δχ*^*2*^ would be systematically affected by the sample size, so the model comparison still needs to refer to other fitting indicators Change [73]. It could be seen that the degree of improvement before and after *CFI*, *NFI* and *RMSEA* does not exceed 0.05, indicating that the model fitting was not significantly improved after adding the common law factor [74]. According to the results of the above two analysis methods, it could be determined that the homology variance problem did not have a serious impact on this study.

### Reliability and validity test of scale

Table 1 shows the fit indexes of the second-order CFA model test and reliability coefficients of the three scales used in the study. The results demonstration that the construct validity and internal consistency reliability of the three scales are within the acceptable range.

**Table 1:** The second-order CFA model analysis and reliability analysis of the scale

Scale	*χ2 /df*	*p*	*CFI*	*GFI*	*NFI*	*TLI*	*RMSEA*	Cronbach’s α
Basic Empathy Scale	2.17	< 0.001	0.85	0.91	0.86	0.58	0.07	0.83
Connor-Davidson Resilience Scale	2.66	< 0.001	0.91	0.93	0.91	0.68	0.06	0.91
Learning Burnout Scale	5.91	< 0.001	0.77	0.75	0.74	0.68	0.09	0.84

### Descriptive statistics for the variables under study and the zero-order correlations among them

Table 2 shows the main descriptive statistics for the variables under study and the zero-order correlations among them.

**Table 2:** Main descriptive statistics and zero-order correlations of variables under study

	Min	Max	*M (SD)*	Skewness	1	2	3	4	5	6	7	8	9
(1) Cognitive empathy	2.25	5.00	3.76 (0.57)	0.01	-								
(2) Emotional empathy	1.13	5.00	3.41 (0.62)	-0.36	0.32**	-							
(3) Tenacity	0.92	4.00	2.46 (0.50)	0.13	0.20**	-0.05	-						
(4) Strength	1.13	4.00	2.70 (0.49)	-0.01	0.32**	0.02	0.76**	-					
(5) Optimism	1.25	4.00	2.46 (0.58)	0.40	0.20**	-0.08	0.53**	0.61**	-				
(6) Dejection	1.00	4.63	2.69 (0.63)	-0.08	-0.26**	-0.15**	-0.26**	-0.29**	-0.07	-			
(7) Improper behavior strength	1.33	4.83	2.89 (0.57)	0.24	-0.10*	-0.06	-0.28**	-0.23**	-0.13**	0.55**	-		
(8) Reduced personal accomplishment	1.17	4.83	2.77 (0.56)	0.16	-0.29**	0.02	-0.46**	-0.44**	-0.38**	0.22**	0.29**	-	
(9) FSES	-6.49	9.18	0.01 (3.59)	0.75	0.17**	0.00	0.13**	0.18**	0.24**	-0.09*	-0.19**	-0.19**	-

^*^p < 0.05, **p < 0.01, ***p < 0.001

The correlational analysis revealed that cognitive empathy positively correlated with resilience (with all factors). Regarding the association between empathy and learning burnout, cognitive empathy was positively correlated with all factors of learning burnout and emotional empathy was negatively correlated with dejection (*r* = -0.15, *p* < 0.01). Finally, in addition to the optimism dimension and dejection dimension, the measures of resilience were negatively associated with all dimensions of learning burnout, with mean correlations ranging between − 0.46 and − 0.07. The variable FSES was correlated (with varying patterns and magnitude of effect sizes) with all the key research variables except emotional empathy.

### The SEM analysis results

**Fig. 2:**
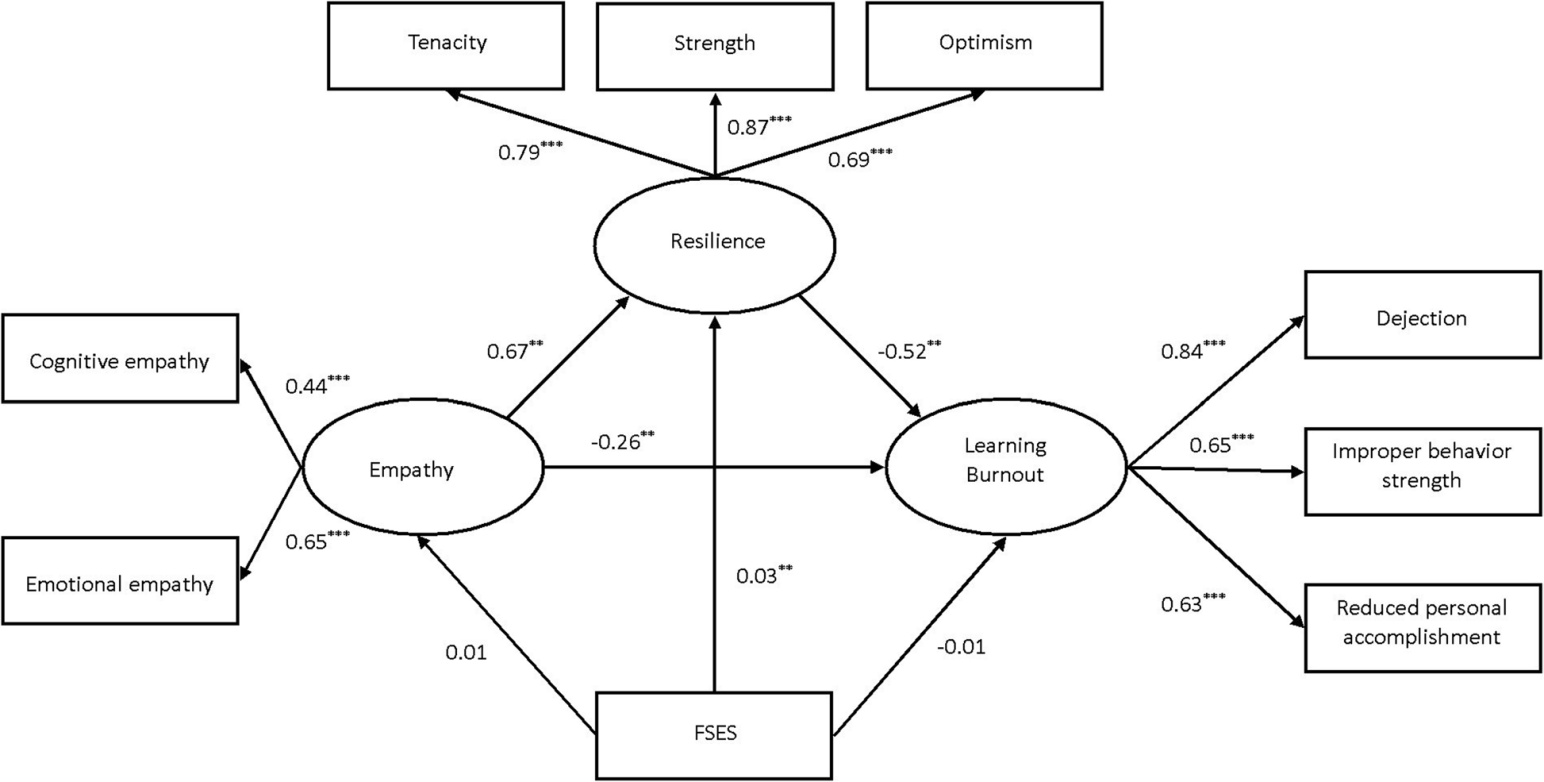
Results of the Structural equation model (SEM). Standardized direct effects were reported

The SEM analysis (see Fig. 2) suggested that the proposed structural model provided an excellent fit for the data: *χ*^*2*^* /df* = 4.58, *p* < 0.001, *RMSEA* = 0.08, *NFI* = 0.95*, NNFI* = 0.91; *CFI* = 0.96, *SRMR* = 0.04. Reading the figure from left to right, we see that empathy exerted a direct, negative, medium-sized standardized effect on learning burnout (*β* = -0.61, *p* < 0.01, 95% C.I. [-0.78 – -0.52]). Concerning the association between empathy and resilience, the standardized direct effect (*β* = 0.67, *p* < 0.01, 95% C.I. [0.47 – 0.88]) was positive and medium in size.

Finally, a large, statistically significant, negative direct standardized effect (*β* = -0.52, *p* < 0.01, 95% C.I. [-0.72 – -0.37]) was found between resilience and learning burnout. Empathy had a significant direct effect on learning burnout, which still existed when the intermediary variable was added (*β* = -0.26, *p* < 0.01, 95% C.I. [-0.47 – -0.09]). Analysis of the indirect effect of empathy on burnout via resilience (*β* = -0.35, *p* < 0.01, 95% C.I. [-0.58 – -0.22]) suggests the crucial importance of having strong resilience for medical college students. Besides, FSES was directly and negatively associated with resilience (*β* = 0.03, *p* < 0.05, 95% C.I. [0.02 – 0.04]), with higher FSES students reporting stronger resilience than lower FSES ones.

## Discussions

In the current study, we investigated the effects of medical college students’ empathy ability on their levels of learning burnout, while also considering the role of resilience. The main findings were as follows. First, medical college students’ empathy ability and their levels of learning burnout were negatively correlated; second, medical college students’ empathy ability was significantly positively associated with resilience, and resilience was significantly negatively associated with school burnout; third, empathy had an indirect effect on medical college students’ learning burnout via resilience; fourth, students’ FSES also exerted significant effects on resilience. We now discuss each of these findings in detail.

The outcome provides support for H1, implying that more empathy medical students were more likely to experience lower levels of learning burnout, which is consistent with previous research results that cultivating empathy is beneficial to alleviating the learning burnout of medical students [1]. This may be because medical students with higher empathy ability having a strong level of self-awareness or self-monitoring are better able to recognize and understand their own emotions and thoughts, and at the same time be good at understanding the experiences of others and maintaining their own unique views on things, and then use these feelings to adjust their emotions to cope with stress, thereby reducing learning burnout [23]. Moreover, some researchers pointed out that empathic reading can better realize deep learning in the process of subject teaching [75], which is conducive to the improvement of academic performance and further relieves learning burnout.

The outcome of the present research bore out H2, suggesting that stronger empathy was associated with more powerful resilience, in other words, the improvement of empathy ability is also conducive to the construction of medical students' psychological resilience [37]. The cultivation of clinical medical students’ cognitive empathy and emotional empathy can encourage them to consider problems from the perspective of others, reduce the infection of others to their own negative emotions and empathy pressure, and improve psychological resilience [39]. Resilience can relieve students' pressure on learning, enhance students' psychological resources and adaptability, and reduce learning burnout, which proves H3. In addition, there is a significant positive correlation between psychological resilience and learning input [76]. Studies have pointed out that psychological resilience has a significant negative predictive effect on academic frustration through core literacy, which is an important cause of academic burnout [2, 77]. When an individual perceives a setback, its protective mechanism is activated to produce avoidance behavior, and the improvement of psychological resilience can make the individual better cope with the setback.

The results of present research confirmed the H4 which show that mental resilience as an internal psychological trait will be positively affected by the individual's empathy ability and then alleviate medical students' learning burnout. Previous studies have also found that emotional intelligence (including empathy) can promote mental resilience [78, 79]. This result also supports the framework of resilience in action. Resilience can alleviate the impact of stress on anxiety and depression [80], enabling individuals to quickly and effectively "rebound" from painful experiences. Individuals with high mental resilience can show faster cardiovascular recovery from negative emotional arousal, have a higher level of positive emotions, a more optimistic attitude towards life, and greater openness [81]. When students are in a positive emotional state, they can be willing to learn, thereby avoiding learning burnout [82]. Medical students having learning burnout experience negative emotions such as depression, anxiety, indifference, exhaustion, confusion, etc. [7, 8]. They can increase the repeated experience of positive emotions through the cultivation of empathy and the improvement of resilience, and inhibit the autonomous response of negative emotions, thereby reducing learning burnout.

Finally, concerning the effects of FSES, resilience was found to increase over the FSES, while learning burnout declined. This is consistent with reports in the literature that students with high family SES can get more company and support, have stronger ability to cope with pressure and higher psychological resilience [53, 83]. Conversely, students who grow up under low family SES will experience more low self-esteem and helplessness due to the lack of parental companionship and inadequate material conditions, show more obvious resistance to stress, and lower psychological resilience [84].

## Implications

The survey results show that medical students’ empathy can negatively predict learning burnout, and psychological resilience has an mediating effect between medical students’ empathy and learning burnout. Thus, we believe cultivating empathy and enhancing psychological flexibility to be effective ways to relieve medical students’ learning burnout and improve their mental health. Accordingly, the following countermeasures are proposed:

### Strengthening professional ethics training for medical students

Medical students should have good ideological and political literacy, professional ethics and medical humanistic spirit, and establish a patient-centered service concept. Medical students should put patients first and firmly establish the service tenet of accepting patients, respecting patients, and caring for patients. Professional ethics education for medical students can not only be carried out through classroom lectures and group discussions, but also through practice to deepen their understanding.

#### Deepening moral education through extracurricular practical activities

Encourage medical students to enter hospitals, make more contact with patients, and provide voluntary services for them; enter communities and rural areas to help people in difficulties. Furthermore, teachers with physician qualifications should organize medical students to conduct free consultations in remote areas and conduct popular science publicity. Through these practical activities, medical students have more contact with patients, understand their thoughts, and gradually establish the concept that treating diseases and saving people is the sacred and glorious duty of doctors [85].

### Deepening professional ethics education in the clinical practice stage

Medical students can transform the theoretical knowledge they have learned into professional skills through internships. The clinical practice stage is also a critical period for medical students to contact the clinic, serve patients, and strengthen professional ethics. Clinical teaching teachers can conduct corresponding professional ethics education for the problems that arise during the internship process, guide medical students to correctly understand the unethical phenomena around them, strengthen the people-oriented and patient-centered concept, and enable medical students to gradually establish correct medical ethics.

### Improving teaching methods

Gradually change the status quo of pure theoretical teaching, encourage clinicians to participate in the teaching of humanistic literacy, advocate philosophy, psychology and other humanities teachers to discuss humanistic issues in the medical field with clinicians, and establish medical humanities teachers’ team who can participate in the entire process of medical education. In the basic course study, try to use a variety of teaching methods such as group discussion, role-playing, psychodrama, etc., so that medical students can slowly understand how to listen, how to think in different places, and so on, so as to better understand patients’ own feelings and the emotional state of others, and finally achieve people-oriented, respect and accept others. In clinical practice, medical students can flexibly use the basic knowledge they have learned through the establishment of doctor-patient relationship, medical history collection, treatment plan negotiation, disease notification and other links [86] to know patients. Thinking and worrying about patients' worries, so that patients can experience the care and respect of doctors in the medical process, and try to avoid medical disputes caused by medical attitudes.

### Improving the overall quality of clinical teachers

In the clinical practice stage, clinical teachers are not only busy with medical work and scientific research, but also take into account the training of medical students' professional skills, and they will pay less attention to the humanistic practice ability of medical students. However, the success of education depends largely on teachers [87]. Teachers play a very important role in the cultivation of empathy for medical students. Teachers must not only improve their communication skills and empathy with patients through various channels, but also guide all teaching activities, and have a subtle influence on students through their own behavior. Teachers can teach medical students how to empathize with patients and establish good communication relationships while dealing with patients’ diseases, and allow medical students to communicate with patients more. In response to common problems encountered by students, students can repeat practice and practice through special topics.

### Paying attention to the psychological resilience level of medical students

Take effective intervention measures to improve the level of psychological resilience. Studies have shown that the use of positive psychology-oriented group counseling can significantly improve the psychological resilience of medical students. Peng has effectively improved different levels of psychological resilience of military medical students through psychological resilience training [88]. In addition, educators can also guide medical students to combine their personal characteristics with family and social support, and guide them to adopt active coping methods to deal with problems in study, interpersonal communication, employment and advancement, and help medical students adjust their psychological state in time and adapt environmental changes so as to increase the level of psychological resilience.

## Limitations and future directions

This study is not without its limitations. First of all, due to space and time constraints, this research adopts a cross-sectional research design. Although previous research has provided the basis for this research, it is still difficult to determine the causal relationship between variables. Future research can adopt longitudinal or experimental research to validate the results of this study. Secondly, due to the large number of variables involved, the sample size of this study is not large enough. Only a medical school in Yunnan Province was used as an example, and the graduate students and undergraduate students were not analyzed separately, resulting in poor age homogeneity of the samples. Future research needs to use a larger sample of medical students to examine the consistency of the research conclusions in different age groups. What’s more, although this study has carried out a Post-hoc Statistical Efficacy Test has been conducted. Nevertheless, an a-priori power analysis is the more modern application that should be done before the samples are collected. Future studies should expand the sample size to enhance the external validity of the results and determine the sample size on the basis of a priori power analysis before participators recruitment. Furthermore, the measures proposed in this study to cultivate medical students' empathy ability and psychological resilience are more derived from speculation, and it is necessary to further examine the effects of these methods in future research.

## Data Availability

The datasets used and analyzed during the current study are available from the corresponding author on reasonable request.
